# Guiding the Self-Organization of Cyber-Physical Systems

**DOI:** 10.3389/frobt.2020.00041

**Published:** 2020-04-03

**Authors:** Carlos Gershenson

**Affiliations:** ^1^Departamento de Ciencias de la Computación, Instituto de Investigaciones en Matemáticas Aplicadas y en Sistemas, Universidad Nacional Autónoma de México, Mexico City, Mexico; ^2^Centro de Ciencias de la Complejidad, Universidad Nacional Autónoma de México, Mexico City, Mexico; ^3^ITMO University, St Petersburg, Russia

**Keywords:** complexity, self-organization, information, adaptation, robustness, antifragility

## Abstract

Self-organization offers a promising approach for designing adaptive systems. Given the inherent complexity of most cyber-physical systems, adaptivity is desired, as predictability is limited. Here I summarize different concepts and approaches that can facilitate self-organization in cyber-physical systems, and thus be exploited for design. Then I mention real-world examples of systems where self-organization has managed to provide solutions that outperform classical approaches, in particular related to urban mobility. Finally, I identify when a centralized, distributed, or self-organizing control is more appropriate.

## 1. Introduction

We are submerged in **complexity**. And this complexity is increasing. But what is complexity? There are dozens of definitions and measures in the literature (Lloyd, 2001; Gershenson and Heylighen, 2005), but not a definite one. Well, life is not properly defined either, and it is not a hindrance for biology. Still, to have an idea of what we refer to, let us go to its etymological root. Complexity comes from the Latin *plexus*, which means entwined. In other words, something complex is difficult to separate. This is because the interactions among its components are relevant (Gershenson, 2013b). Relevant because they co-determine the future of the system. Thus, if we do not consider such interactions, but study components in isolation, we will not be able to understand the system properly. Also, interactions can generate novel information, not present in initial nor boundary conditions. This novel information limits predictability (Gershenson, 2013a) and is the source of computational irreducibility (Wolfram, 2002), i.e., there is no shortcut to know the future: one must go through all intermediate steps, because the information produced in the process is required to reach/compute the future.

A recent collaborative effort produced this definition: “Complexity science, also called complex systems science, studies how a large collection of components—locally interacting with each other at small scales—can spontaneously self-organize to exhibit non-trivial global structures and behaviors at larger scales, often without external intervention, central authorities or leaders. The properties of the collection may not be understood or predicted from the full knowledge of its constituents alone. Such a collection is called a complex system and it requires new mathematical frameworks and scientific methodologies for its investigation.” (De Domenico et al., 2019).

One of the core concepts explained in De Domenico et al. (2019) is **self-organization**: “Interactions between components of a complex system may produce a global pattern or behavior. This is often described as self-organization, as there is no central or external controller. Rather, the “control” of a self-organizing system is distributed across components and integrated through their interactions. Self-organization may produce physical/functional structures like crystalline patterns of materials and morphologies of living organisms, or dynamic/informational behaviors like shoaling behaviors of fish and electrical pulses propagating in animal muscles. As the system becomes more organized by this process, new interaction patterns may emerge over time, potentially leading to the production of greater complexity.” Common examples of self-organizing systems include flocks of birds, schools of fishes, insect swarms, herds, crowds, and other collective phenomena (Camazine et al., 2003; Vicsek and Zafeiris, 2012), although self-organization is not restricted to living systems (Nicolis and Prigogine, 1977; Haken, 1988; Gershenson and Heylighen, 2003; Prokopenko et al., 2009).

There are many cases where self-organization has been used as an approach in **engineering** (Di Marzo Serugendo et al., 2004; De Wolf et al., 2005; Zambonelli and Rana, 2005; Mamei et al., 2006; Helbing et al., 2007; Dressler, 2008; Müller-Schloer et al., 2011; Rohden et al., 2012; Brambilla et al., 2013; Rubenstein et al., 2014; Vásárhelyi et al., 2018). In these cases, we can *describe* a system as self-organizing when elements interact to achieve dynamically a global function or behavior (Gershenson, 2007). In other words, instead of designing directly a solution, one regulates the potential interactions among elements. This is useful in *non-stationary* problems: when the situation changes, then the system adapts by itself. Since interactions in complex systems produce novel information, it is common that this information will change a complex problem. Not only its state, but also its state space. Thus, self-organization can be useful to face complexity by providing general adaptation mechanisms. Several methodologies using self-organization have been proposed (see Frei and Di Marzo Serugendo, 2011 for an overview), although the approach has not been widely applied.

In a parallel effort, **guided self-organization** attempts to combine seemingly opposed processes: design to define and regulate the properties and behavior of a system (one tells the system what to do), and self-organization that implies certain autonomy and adaptability (the system follows its own dynamics) (Prokopenko, 2009, 2014; Ay et al., 2012; Polani et al., 2013). Guided self-organization can be understood as “the steering of the self-organizing dynamics of a system toward a desired configuration” (Gershenson, 2012).

**Figure 1:**
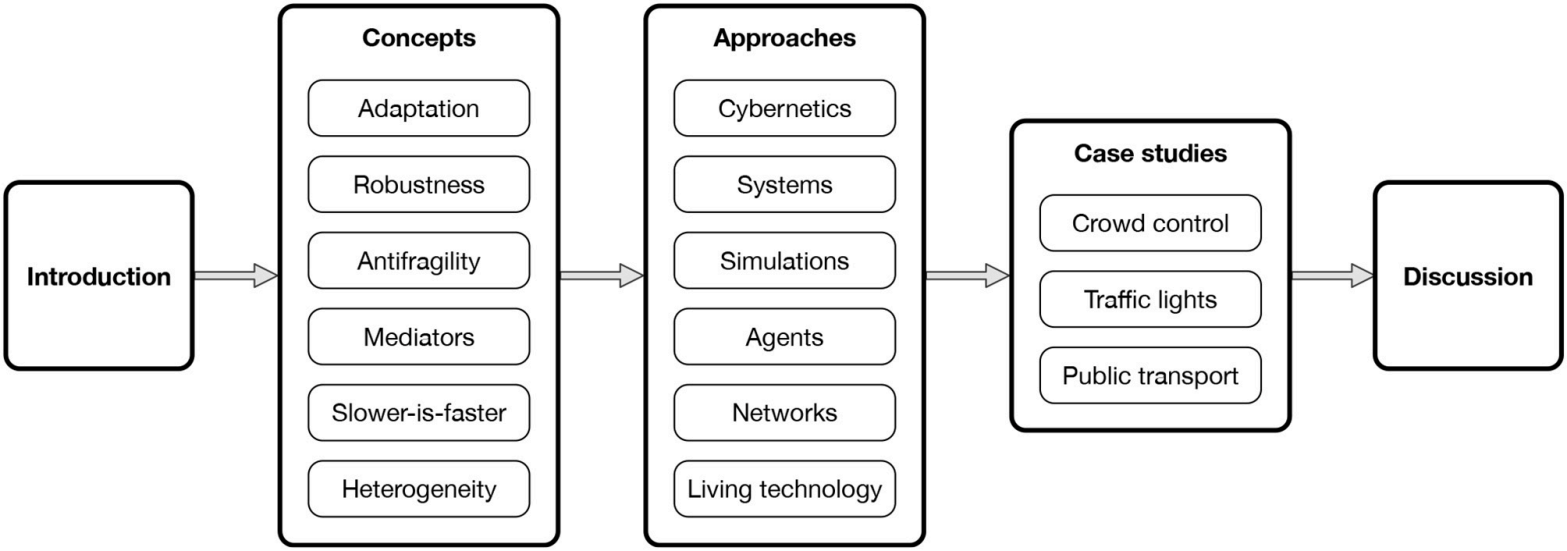
Diagram of the paper structure depicting sections and subsections.

In this paper, I compile concepts and approaches useful for designing self-organizing systems in the physical realm. I illustrate these with case studies from urban mobility before discussing implications. A diagram of the paper structure is shown in Figure 1.

## 2. Concepts

Several concepts are useful to design and guide self-organizing systems. In this section, a non-exhaustive list is presented.

### 2.1. Adaptation

Adaptation can be defined as a change in an agent or system as a response to a state of its environment that will help the agent or system to fulfill its goals (Gershenson, 2007). Living systems naturally adapt to changes in their environment, and artificial systems can benefit from exhibiting adaptation (Holland, 1975; Steels and Brooks, 1995; Bedau et al., 2013).

If problems are **stationary**, i.e., do not change, then it is worthwhile attempting to predict the future of a system to control it. However, for **non-stationary** problems, predictability by definition is limited. Novel information generated by interactions in complex systems can lead to non-stationarity. In this case, adaptation is desirable to complement the unpredictable aspects of a problem (Gershenson, 2013a). And self-organization offers a method for building adaptive systems.

For example, city traffic is changing constantly: every time a red light switches to green, the number of waiting vehicles is different. Thus, the timing of the traffic lights should also change to prevent idling. Traditional adaptive traffic light control methods (e.g., Sydney, Dublin, Singapore) use sensors to shift phases depending on recent average demands. This is usually better than not having adaptation, where the best possible option would be to take average measurements, set fixed phases, and perhaps change the programs a few times per day. However, if traffic lights can adapt at the same timescale as the traffic demand does, i.e., every cycle, then the performance would be much improved (Goel et al., 2017).

Adaptation implies *flexibility* and can take place at different timescales: *learning* is relatively fast, *development* occurs during the lifetime of an individual, and *evolution* acts across generations.

### 2.2. Robustness

A system is **robust** if it continues to function in the face of perturbations (Wagner, 2005), and in general any type of change. As with adaptation, robustness is prevalent in living systems and desirable in artificial ones (Jen, 2005).

Robustness and adaptability are complementary: a system has to be robust enough to survive while it adapts, and adaptation can favor robustness.

For example, the Internet is quite robust. The TCP/IP protocol was designed to resist nuclear warfare. If any server goes down, other servers will manage to transmit packages, unless the network becomes disconnected. At the structural level (which servers are linked, which pages are linked), self-organization has led to a scale-free topology (Barabási et al., 2000), which is also robust to random failures (although fragile to directed attacks Caldarelli, 2007). This is because only few nodes have several connections, so most probably a random failure will affect a non-important node. However, directed attacks can aim for the hubs.

Robust systems are more prone to be *scalable* than fragile ones. Adding new components or functionality to a system can be seen as a type of perturbation, so in this sense robustness becomes a requirement for scalability.

### 2.3. Antifragility

A fragile system is damaged by perturbations. A robust system is unaffected by perturbations. An **antifragile** system *benefits* from perturbations (Taleb, 2012). Particular examples of systems that benefit from noise had been already identified (Atlan, 1974), and the concept of antifragility can be seen as a generalization.

For example, the immune system is antifragile. Children who grow up in extremely sanitized conditions are not exposed to pathogens (perturbations), so their immune systems do not develop, leading to stronger infections and allergies in adulthood. Certainly, children should not be infected intentionally, but being exposed to a “normal” amount of pathogens and falling ill now and then is helpful for training the immune system.

We have recently proposed a measure of antifragility (Pineda et al., 2019), which is positive when perturbations improve the performance of a system, negative when perturbations decrease the performance (fragility), and zero when perturbations do not affect the performance (robustness). An important aspect is that there is no “optimal” antifragility independent of an environment. A system should be as antifragile as its environment varies (this is related with **requisite variety**, discussed in section 3.1).

### 2.4. Mediators

Interactions can be classified as positive, neutral, or negative, depending on the effect they have on the goals of a system (Gershenson, 2007, 2011b).

A **mediator** arbitrates among the elements of a system, to minimize conflict, interferences and frictions (negative interactions); and to maximize cooperation and synergy (positive interactions) (Michod, 2003; Heylighen, 2006; Gershenson, 2007).

Negative interactions, by definition, are those that prevent or damage the functionality, performance, goals, or behavior of a system. Positive interactions would benefit, facilitate, or promote them. Neutral interactions do not affect them. For example, actions that generate a cost but fail to provide a benefit for a society can be said to generate friction, e.g., aggression. If the benefit provided by actions is greater than the cost, one can say that they are synergistic, e.g., politeness. If the cost and benefit balance out, the interactions would be neutral, e.g., tolerance.

Traffic rules can be seen as examples of mediators. They aim at reducing conflict in urban mobility. Without these rules, we would need to decide constantly on which side of the streets to drive, how to give way, make turns, etc. Even when rules and norms vary from country to country, and in some cases from city to city, when everybody follows the same set of rules (mediators), conflicts tend to be reduced.

Money is another example. It mediates transactions that are much facilitated compared to bartering.

Designing mediators can be useful for regulating systems where the elements cannot be modified. Still, mediators can change the interactions between elements, leading to different systemic behavior and properties (see case study in section 4.1).

### 2.5. Slower-Is-Faster Effect

Probably this effect was first described about 20 years ago while modeling crowd dynamics (Helbing et al., 2000a,b). If people trying to evacuate a room are panicked (trying to exit faster), then they create friction (negative interactions) that leads to a “turbulent” flow that is slower than if people exit calmly (neutral interactions), thus with a “laminar” flow. The same effect has been studied in vehicular traffic, logistics, public transport, social dynamics, ecological systems, and adaptive processes (Gershenson and Helbing, 2015).

In general, the slower-is-faster effect occurs when a system performs worse as its components try to do better. This implies that a balance between doing “too few” and doing “too much” is necessary. However, in many cases this balance is dynamic, as with antifragility. For example, the optimal speed for highway traffic (that maximizes flow) depends on the vehicular density. For this reason, systems that present a slower-is-faster effect, require constant adaptation, that can be achieved through self-organization.

The slower-is-faster effect may refer to any variable, not only speed. For example, growth or profits are not necessarily maximized in the long term with a short-term maximization strategy. Managing natural resources, such as fisheries, requires this understanding: if all resources are depleted, then in the near future there will be no profits. Maximizing profits requires a careful balance between short-term action and long-term planning. As with the case of highway traffic, usually this balance is non-stationary.

### 2.6. Heterogeneity

Most of our models of complex systems are homogeneous: all components have the same properties. This simplification is useful when we face computational limitations. However, increasing processing power and data availability have allowed us to make more realistic models, where different elements of a system have varying properties.

Perhaps the most studied heterogeneity in complex systems is the one of network topologies (Albert and Barabási, 2002; Newman et al., 2006; Gershenson and Prokopenko, 2011; Barabási, 2016) (see section 3.5). Many networks are heterogeneous, with few elements having lots of connections and many elements having few connections. This leads to important differences with homogeneous, regular networks, where all elements have the same number of connections. Apart from the robustness already mentioned, heterogeneous networks can also transmit information faster (they have shorter average path lengths) (Aldana, 2003).

More recently, temporal heterogeneity has been also studied (Cocho et al., 2015; Morales et al., 2018), i.e., systems where different components change at different rates. In a similar way to structural heterogeneity, few elements change slower than most elements. This heterogeneity seems to lead to a balance where slow elements are robust and fast elements are adaptable. In homogeneous systems, this balance is achieved only in phase transitions, which can be characterized as “critical” (Balleza et al., 2008). However, heterogeneity seems to expand the balance beyond criticality, making it easier to search an unknown parameter space, simply because different components diversify any search procedure (Martínez-Arévalo et al., in preparation).

## 3. Approaches

How to implement the properties related to self-organization in cyber-physical systems? The concept of self-organizing systems originated within cybernetics (Ashby, 1947, 1962; von Foerster, 1960; Heylighen et al., 1993), where useful approaches were already developed.

### 3.1. Cybernetics

Ashby not only coined the term “self-organizing system,” but he also proposed the law of **requisite variety** (Ashby, 1956; Heylighen and Joslyn, 2001; Bar-Yam, 2004; Gershenson, 2015). Variety can be understood as the possible number of states that a system can have. This law states that an *active* controller must have at least as much variety as the system it is trying to control. For example, if we want a robot at a manufacturing plant to deal with seven different types of boxes, then it should be able to distinguish and make the appropriate decisions to handle each type of box. A common problem is that complexity explodes variety and vice versa. Therefore, traditional (non-adaptive) approaches become limited. To handle the variety of a system, we can either reduce its variety (using mediators), or increase the variety of the controller, but then the latter will imply an increase in the complexity of the controller as well.

Everything else being equal, the variety of *non-stationary* domains will be greater or equal than those of *stationary* ones, as their change usually implies a greater number of potential states. Therefore, **adaptive** controllers and **antifragile** mechanisms have to consider this increased variety.

*Active* controllers are related with feedforward and feedback (positive or negative) control. Feedback occurs in response to a signal or perturbation, so it can be seen as a type of **adaptation** (Gershenson, 2007). Negative feedback reduces the effect of the perturbation, trying to reach stability, while positive feedback amplifies perturbations, leading to greater change. Feedforward control might be preferred, as it acts on a perturbation or signal before it can affect the controlled. However, this requires *anticipation*, and since complexity implies a limited predictability due to novel information being generated by relevant interactions (non-stationarity), this type of control will also be limited.

**Figure 2:**
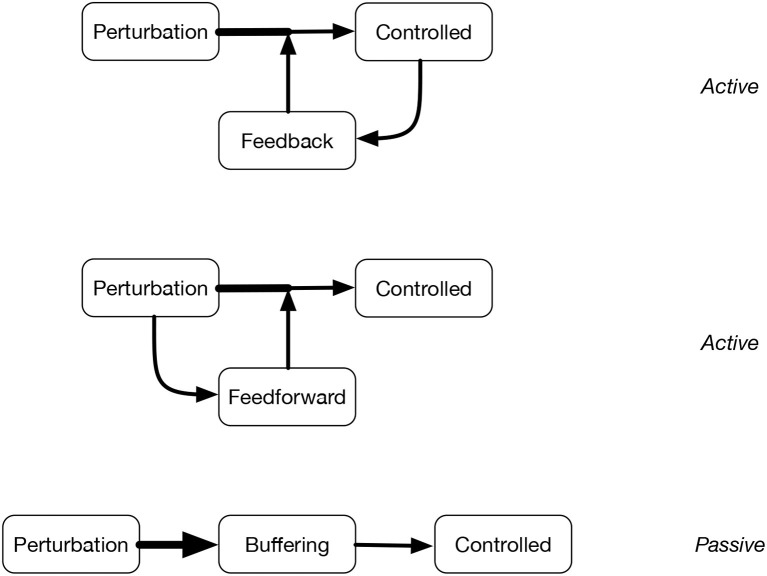
Diagrams of feedback control, feedforward control, and buffering. In different ways, they try to reduce or eliminate the effect of perturbations on the controlled, either actively or passively. Arrows indicate the effects of perturbations: wider lines indicate greater effects. Ideally, the control mechanisms should be able to eliminate completely the effect of perturbations.

Complementary to active controllers, *passive* controllers were also studied in cybernetics, related to buffering. Passive control can increase the **robustness** of systems, since it prevents perturbations from affecting the controlled. Figure 2 illustrates active and passive controllers.

There is an interesting relationship between variety and **heterogeneity**. Heterogenous systems by definition have more variety, so in principle they should be able to control more situations than similar homogeneous systems. However, they might be less robust and more complicated to design and understand. For example, “if there is a system of ten agents each able to solve ten tasks, a homogeneous system will be able to solve ten tasks robustly (if we do not consider combinations as new tasks). A fully heterogeneous system would be able to solve a hundred tasks, but it would be fragile if one agent failed.” (Gershenson, 2007, p. 53). In this case, the homogeneous system would be **robust**, because if one agent fails, others can perform the same function. Still, the **variety** of the system would be restricted to ten tasks. The heterogeneous system would have a tenfold variety, but if a single agent fails, then no other agent would be able to take over the task, and the system would fail as well. Thus, a balance between homogeneity and heterogeneity should also give us a balance between **robustness** and **adaptability** (Langton, 1990; Kauffman, 1993).

### 3.2. Systems

Contemporary and overlapped with cybernetics, systems theory has also permeated into all disciplines (von Bertalanffy, 1968). The word “system” comes from the ancient Greek $\sigma \overset{´}{\upsilon }\sigma \tau \epsilon \mu \alpha$ (sýstema), which means a whole made of several parts. It is a useful abstraction that can be applied to describe several phenomena at different scales. Moreover, it can be the basis for understanding how elements interact to generate behavior or properties at the system level, and how these properties regulate or constrain the behavior or properties of the elements.

Cybernetics and systems theory naturally merge in cyber-physical systems, where control and communication are required in the understanding and engineering of systems composed of “bits and atoms,” i.e., digital information is entwined with physical mechanisms.

In a similar way, cyber-social systems are those that merge digital technology and social interactions. The “human factor” increases the **variety** of such systems, and our “creativity” limits even more their predictability.

### 3.3. Simulations

We can consider computers as telescopes of **complexity** (Pagels, 1989). In other words, without computers, our cognitive abilities are limited to studying models considering not many more than two or three variables. To explore models with thousands or millions of variables, **computer simulations** are necessary (Gershenson, 2007) because of computational irreducibility (Wolfram, 2002). Complexity implies that new information is generated by interactions, so there is no “shortcut” to the future and all intermediate steps are necessary (Wuensche and Lesser, 1992). This limits inherently the predictability of systems (Gershenson, 2013a).

Simulations do not replace other approaches, but their usefulness can be seen in the spreading of computational methods to all disciplines.

Also, simulations allow us to contrast theories in a synthetic way (Steels, 1993). The inductive method validates theories through observation of phenomena.The synthetic method builds artificial systems based on a theory, and then this is validated observing the performance of the artificial system (Simon, 1996).

Since one can contrast different theories using computer simulations, it can be said that computational social sciences are “hardening” the social sciences (Axelrod, 1997; Lazer et al., 2009).

### 3.4. Agents

**Agent-based modeling** (Bonabeau, 2002; Schweitzer, 2003; Epstein, 2006; Wilensky and Rand, 2015) has been a useful approach to describe complex systems. An agent can be defined as an entity that *acts* on its environment (Gershenson, 2007). As such, they can be used to model *active* controllers.

Agents have been used to model cognitive systems of different flavors, including rational (Wooldridge and Jennings, 1995), adaptive (Maes, 1994), social (Epstein and Axtell, 1996; Gershenson, 2001), and economic (Arthur, 1999; Challet et al., 2013).

Considering elements of a complex systems as agents, with states, goals, and rules allows us to study how changes at one scale lead to effects at another scale. The effects can go in both directions: changes in agents leading to changes in the system and vice versa. Moreover, systems can also be described as (higher scale) agents.

Another advantage of agent-based modeling is that such models are closer to common language than previous modeling approaches based in e.g., differential equations. Therefore, people do not require a strong mathematical background to develop models using a multi-agent approach.

### 3.5. Networks

Another approach that is becoming more and more popular as data availability and computing power increase is **network science** (Newman, 2003; Newman et al., 2006; Barabási, 2016). Networks have the benefit of being able to represent naturally elements (nodes) and interactions (links). The relationship between the structure and function of networks has been an intense area of study, where self-organization can play a relevant role (Gershenson, 2012).

Different organizations of the same elements can lead to radically different functionalities. A classical example is different arrangements (allotropes) of carbon atoms, which can lead to charcoal, diamond, graphite, graphene, nanotubes, buckyballs, etc. The components are the same, but changing their organization (structure) leads to radically different properties (function) of these materials.

The **robustness** of systems can be promoted through different mechanisms (Gershenson, 2012), such as redundancy (having several copies of the same element), degeneracy (having different elements perform the same function), modularity (short-range links stronger than long-range ones), and scale-free-like (heterogeneous) topologies (few elements with several links, several elements with few links).

### 3.6. Living Technology

Ethology—the study of animal behavior—has been taken as an inspiration to build **adaptive** systems (Beer, 1990; Maes, 1994; Steels and Brooks, 1995) and to study complex artificial systems (Rahwan et al., 2019). Animals have evolved to survive in complex environments, so adaptive strategies and self-organizing mechanisms found in nature have been used in cyber-physical systems. In this sense, **living technology** (Bedau et al., 2009; Gershenson et al., 2018) takes the advantageous properties of living systems and applies them in socio-technical systems, from protocells (Rasmussen et al., 2008) to cities (Gershenson, 2013c).

Living technology has been defined as technology that exhibits the properties of living systems, such as **adaptation**, learning, evolvability, **robustness**, and self-organization. First-order living technology is actually alive, either manipulating existing living systems (Gibson et al., 2010; Kriegman et al., 2020) or (eventually) building them from scratch (Rasmussen et al., 2008; Čejková et al., 2017). Second-order living technology uses living systems as components to achieve the desired properties found in living systems (Benyus, 1997; Liu and Tsui, 2006).

## 4. Case Studies

In this section, I illustrate the previous concepts and approaches with case studies we have worked with in recent years, related to urban mobility. Particular concepts are highlighted, although approaches are implicitly used.

### 4.1. Crowd Control

More than a hundred million people use the hundred busiest metro systems in the world every day, a number that is growing fast as the urban population is increasing and cities develop. In the Mexico City Metro and other **cyber-social systems**, people would normally push each other, not letting passengers exit trains, collapsing the systems. How to regulate passenger behavior, when a selfish approach might seem to bring individual benefit but lead to collective inefficiency? One can think of different **mediators**, but they can be costly to try in real systems. To explore alternatives, we first used **simulations** of a model of crowd dynamics (Helbing et al., 2000a) and then implemented a pilot study in the Balderas station of the Mexico City Metro on December, 2016 (Carreón et al., 2017). The pilot was a success and it has since been extended to several other busy stations.

**Figure 3:**
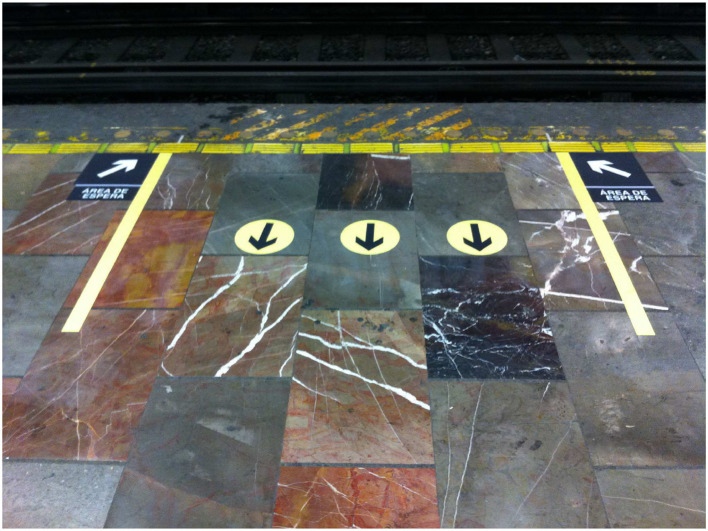
Signs installed to mediate passenger boarding and descent in Mexico City Metro. Reproduced from Carreón et al. (2017) under the Creative Commons CCBY license https://doi.org/10.1371/journal.pone.0190100.g015.

**Figure 4:**
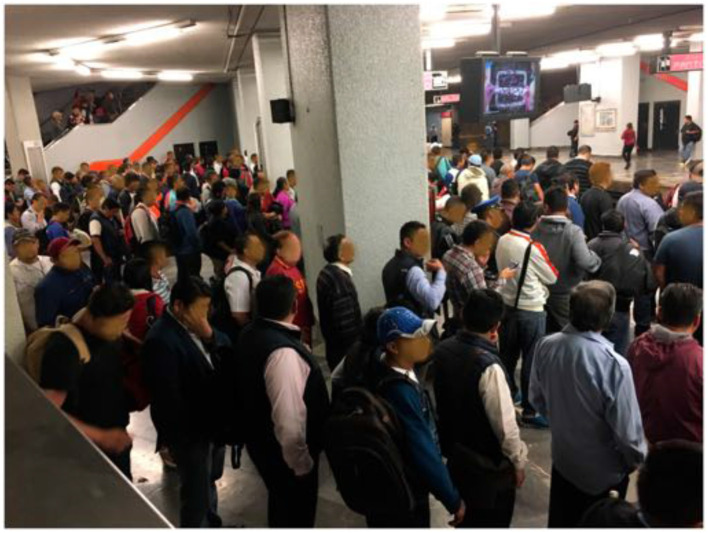
Passengers queuing waiting for a train in Mexico City Metro during rush hour, San Lázaro metro station. Reproduced from Carreón et al. (2017) under the Creative Commons CCBY license https://doi.org/10.1371/journal.pone.0190100.g016.

The intervention consisted of “simple” signs that indicate passengers roughly where the train doors will be, asking them to leave free space for exiting passengers, as shown in Figure 3. What we did not expect nor suggest was that people would queue (Figure 4), and that these queues could even go upstairs as people respected them.

This intervention managed to change the behavior of the passengers and thus the crowd, without changing the elements of the system (where could we get different “educated” passengers from?). The signs **mediated** *interactions* between people. This is an example of a **passive** control, where interactions are regulated “simply” providing useful information. The mediators managed to change the *structure* of the crowd, leading to a more efficient *function*.

### 4.2. Traffic Light Coordination

The coordination of traffic lights is an EXP-complete problem, meaning that in theory it takes exponentially more time to find a solution as more intersections are added to a street network. Also, the precise number of vehicles changes every cycle, so in practice the problem changes faster than it can be optimized. An **active** controller should **adapt** as fast as the controlled changes (**requisite temporal variety**), and for that sensors are required to provide relevant information to the controller.

With this in mind, we have proposed self-organizing algorithms that can coordinate traffic flows and adapt to constant changes in the demand as fast as it changes (Gershenson, 2005; Zapotecatl et al., 2017), achieving close-to-optimal performance (Gershenson and Rosenblueth, 2012). The main idea behind the algorithms is that streets with a higher demand get a preference. This is implemented by counting how many vehicles are approaching or waiting behind red lights, and when the integral over time of this counter reaches a threshold, then the green light is requested. Thus, busier directions will wait less for a green light. This increases the probability that vehicles will aggregate behind red lights with few cars, leading to the formation of platoons. As platoons reach a certain size, they can request a green light before they even reach an intersection (because they quickly reach the threshold), so vehicles do not need to stop, unless there are other vehicles or pedestrians crossing. Platoons are easier to coordinate than individual vehicles, as they leave spaces between them that other platoons can use without interference. When densities are high, the preference is given to the street that has more space after the intersection, preventing gridlocks.

**Figure 5:**
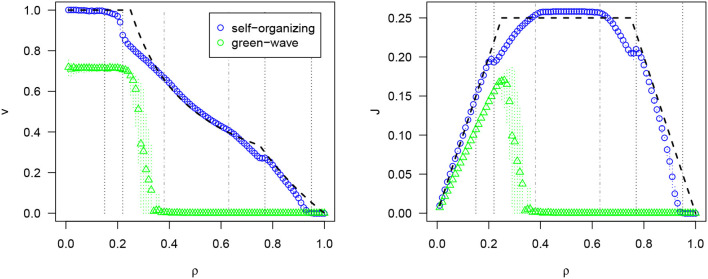
Results of self-organizing traffic lights: average velocity *v* and average flux *J* for different densities ρ. Optimality curves shown with dashed black lines. Reproduced from Zubillaga et al. (2014) under the Creative Commons Attribution License.

It is difficult to compare the performance of self-organizing traffic lights, as there are no benchmarks in traffic light coordination. However, they are close to optimal. We can define optimality by calculating the maximum performance (measured in terms of velocity or flow) of isolated intersections for different densities. If a **system** with several intersections performs as efficient at every intersection, we can say that the coordination is optimal. Figure 5 shows a comparison of the self-organizing approach and a traditional top-down control method known as the “green wave” that attempts to offset phases according to the expected speed of vehicles. However, demands change constantly and this method cannot adapt, leading to gridlocks even at medium densities. The self-organizing method achieves optimality for low densities (no vehicle stops) and medium densities (all intersections are used at maximum capacity: there are always vehicles crossing all intersections. Topologically it is not possible to improve this). For other densities, the performance is close to the optimality curves (for details, see Gershenson and Rosenblueth, 2012).

More recently, we have found that self-organizing traffic lights would improve traffic more than if all vehicles were autonomous but with traditional traffic lights. Nevertheless, autonomous vehicles and self-organizing traffic lights are even better (Zapotecatl, 2019).

By distributing control locally, the **requisite variety** of the traffic light coordination can be tackled **robustly** as conditions change, while the formation of platoons **self-organizes** the traffic flows and assists the coordination of intersection controllers at the city scale. In this way, the traffic lights are **mediators** of vehicles, but the vehicles are also **mediators** of traffic lights. We have made simulations with up to ten thousand intersections achieving efficient or optimal coordination, so this solution is certainly scalable.

As there are so many variables involved in this system, **simulations** are necessary to explore and test potential solutions. It is natural to represent the topology of a city as a **network**, where nodes are intersections and links are streets connecting them. Vehicles and traffic lights can be usefully described as **agents**, since they act on their environment. It is worth noting that then traffic lights become part of the environment of vehicles, while vehicles are part of the environment of traffic lights.

### 4.3. Public Transport Regulation

In theory, passengers in public transport are served optimally when vehicle headway—the time between arrivals at a station—is equal. However, as we have shown, an equal headway configuration is unstable by nature (Gershenson and Pineda, 2009), since delays become amplified by positive feedbacks. Thus, many efforts have been made by transportation engineers to prevent the “equal headway instability,” also known as the “bus bunching problem.”

To keep equal headways, all vehicles—trains, trams, buses—must wait the same time at each station. This time can vary from station to station, but it must be fixed or some vehicles will go faster than others, leading to unequal headways and potentially to the collapse of the **system**. Since the precise number of passengers varies each time a vehicle reaches a station, and thus the required waiting time, then either vehicles will require a margin and be idle, or they will depart before servicing all passengers when these are more than expected.

We proposed a self-organizing algorithm inspired by ant colony communication (Gershenson, 2011a; Carreón et al., 2017), so this can be seen as an example of **living technology**. Some ant species communicate via their environment, a phenomenon known as stigmergy (Theraulaz and Bonabeau, 1999). When they find a food source, they return to their nest leaving a pheromone trail. This indicates the food location to other ants. When they find the food, they can reinforce the trail while returning to their nest. Since pheromones evaporate, once the food is finished, ants stop reinforcing the trail, and they start exploring again. In the case of our algorithm, vehicles can be seen as ants, and we wanted a pheromone-like environmental signal to be used to indicate when the last vehicle had passed. However, pheromones reduce their concentration, while we needed an increasing signal, so we defined “antipheromones” that are secreted by the environment, increase their concentration in time, and are erased by vehicles as they pass.

In our algorithm, each vehicle “simply” tries to keep equal distance to the vehicles in front and behind (using antipheromones as **mediators**), but is flexible enough to serve passengers at stations and at the same time prevent idling. Equal headways are not maintained, but the system does not collapse. Rather, its performance is even better than the case with equal headways, i.e., it is supraoptimal. This is because of the **slower-is-faster effect**: It is true that passengers minimize their waiting time at stations with equal headways (as expected by theory). But their total travel time is not independent of the equal headways, so idling will increase their total travel time. With the self-organizing algorithm, passengers wait more at stations, but once they board a vehicle, they will reach their destination faster, as there is no idling. Again, **adaptation** takes place at the scales at which the system changes. We can say that this approach is **antifragile**, as supraoptimality is achieved precisely because of the “noise.” (**heterogeneity**) of arriving passengers. If all stations had always the same demand (homogeneous), then the self-organizing algorithm would perform as good as the theoretical optimum, i.e., less than supraoptimal.

**Figure 6:**
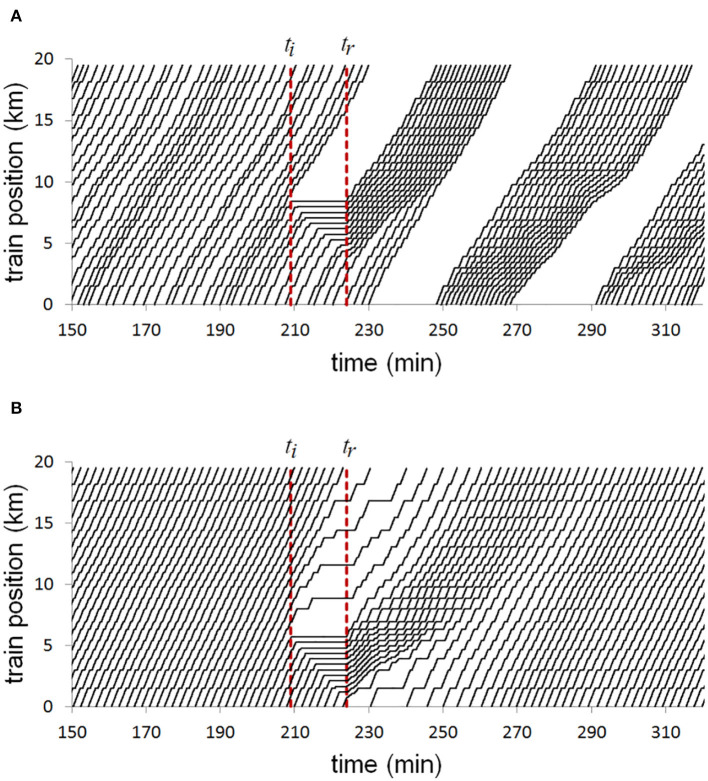
Diagram showing positions of trains at different times. Trains move upwards in distance and rightwards in time. There is an interruption of service at *t*_*i*_, and it is reestablished 15 min later at *t*_*r*_. **(A)** Current control method. **(B)** Self-organizing method. Reproduced from Carreón et al. (2017) under the Creative Commons CCBY license https://doi.org/10.1371/journal.pone.0190100.g014.

Figure 6 shows results from a simulation of Line 1 of the Mexico City Metro. On the top panel, the trajectories of trains using the current regulation method is depicted. There is a 15 min interruption of the service, and it can be seen that the system does not recover. In reality, the system does recover, but it requires human intervention and can take one, two, or more hours, depending on the passenger demand. On the bottom panel of Figure 6, the trajectories of a similar scenario are shown, but using our self-organizing method. It can be seen that even before the service is reestablished, the vehicles try to maintain equal headways with their neighbors, delaying vehicles ahead of the station where service was interrupted. Once service is reestablished, since the intervals between trains did not collapse, trains can quickly **adapt** and respond to the delayed service, recovering a desired configuration in less than half an hour.

## 5. Discussion

We cannot reduce the complexity of several systems we have to deal with. Novel information produced by interactions leads to changes, making problems non-stationary. For example, in the case of traffic lights, one cannot try to optimize intersections in isolation and expect the system to be coordinated. Since the “output” of one intersection becomes the “input” of the next one downstream, this information should be constantly updated by sensors and taken into consideration by controllers.

Self-organization has been used in a broad variety of cyber-physical systems. It allows systems to adapt at the scales at which the problem they are solving changes in a robust fashion. In addition to the case studies mentioned in the previous section, dynamic road pricing in Singapore and variable parking cost in San Francisco are examples of self-organization being used to regulate urban mobility. We can see that the same principles apply in other cyber-physical and cyber-social systems, from telecommunications (Amoretti and Gershenson, 2016) to organizations (Gershenson, 2008).

As in the case of crowd control, there are many systems where we cannot change the components. Still, we can try to mediate interactions to control the function of the system. We will not change politicians. But perhaps we can regulate their interactions to improve politics. We cannot change teachers. But maybe novel mediators can improve education. Businesspeople will not change. But probably promoting certain interactions and restricting others can improve economies. It can take lots of energy to turn charcoal into diamond, but it can be done. They are made of the same atoms. “Only” their organization is different.

A relevant step toward adopting self-organizing controllers is to give up the desire to control completely our systems. This implies accepting that predictability is limited by complexity, and that **adaptation** should complement this inherent uncertainty, even if we do not know how systems will adapt. As complexity limits our predictability, systems require certain autonomy to make the “right decisions.” Even if we use traditional approaches, we do not have full control of our systems, as they are constantly entering unexpected situations. We would like to be able to be sure that our systems will never fail, but they will. We can have formal proofs but these are also limited, since they assume idealized/closed/predefined situations. Self-organizing systems can do the same as traditional engineered systems and more, as they can deal with more realistic/open/variable situations. We just have to (systematically and cautiously) try and see, constantly adapting (Gershenson, 2007). Even if a solution already worked, it does not assure that it will continue working (as conditions change) or that it can be applied in the same way in a different context.

The best solution depends on the context/environment /problem. In some cases, centralized control will be good, in others distributed is more appropriate, in yet others self-organizing. As shown in Table 1, **centralized** control is appropriate when causality should be top-down. Because of the law of requisite variety, systems with a high variety/complexity will require a controller with a high variety/complexity, so the centralized approach becomes less viable. **Distributed** control can deal with a greater complexity, but it is still limited, because the integration of the distributed solutions is not necessarily trivial. This limits distributed control to homogeneous systems: since information flow across the system is restricted, the local solutions assume that each local problem is similar. As illustrated in the traffic lights example, **self-organizing** control can deal with top-down and bottom-up causality (multiscale), as components can interact in a distributed fashion to change system properties (bottom-up), but then the system properties can mediate (top-down) to regulate the behavior of components. Self-organization can be scalable, adaptive, robust, and can deal with a high complexity and homogenous or heterogeneous problems. It is not that one approach is better than others, but they are more appropriate for different problems. Centralized control is easier to implement and understand, but is useful for low complexity/variety problems. Distributed control can deal with a greater complexity, but only for homogeneous, separable systems. Self-organizing systems might be more difficult to design and test, but they can handle greater complexity/variety/diversity.

**Table 1:** Different control approaches are more appropriate for different causalities, complexities, and diversities.

**Control**	**Causality**	**Complexity**	**Diversity**
Centralized	Top-down	Low	Homogeneous or heterogeneous
Distributed	Bottom-up	Medium	Homogeneous
Self-organizing	Multiscale	High	Homogeneous or heterogeneous

How the control is organized is certainly relevant, but also whether the control is active or passive. As shown in Table 2, **active** control is more related with adaptation and antifragility, as these concepts imply constant change in the **function** of the controller. An agent-based approach is natural here, as it is straightforward to describe actions with agents, since these are entities that act on their environment. On the other hand, **passive** control is more related with robustness and heterogeneity, as these are intrinsic properties of systems and their **structure** (independently on whether there is change or not in the environment). A network description is useful in this case, as the relationships between elements can describe the organization of a system. Note that these are not exclusive, e.g., one can certainly use both active and passive controllers, or combine agents represented as networks, or study how structure and function affect each other. Also, the concepts and approaches not mentioned here apply to both control cases. Moreover, the relationship between structure and function is far from trivial and has been an open area of research (Heylighen, 1999), since structure defines function but also function can change structure. In many cases, we design structure for a desired function, but also we can design function for a desired structure (Dorigo et al., 2004; Werfel et al., 2014).

**Table 2:** Different control types are more related to certain concepts, approaches, and aspects.

**Control**	**Concepts**	**Approaches**	**Aspects**
Active	Adaptation/antifragility	Agents	Functional
Passive	Robustness/heterogeneity	Networks	Structural

As the complexity of our cyber-physical systems increases, and also our understanding of it, we will see more self-organizing approaches. Perhaps names will differ, but the concepts presented here are required to control cyber-physical and cyber-social systems by guiding their self-organization.

## Author Contributions

The author confirms being the sole contributor of this work and has approved it for publication.

### Conflict of Interest

The author declares that the research was conducted in the absence of any commercial or financial relationships that could be construed as a potential conflict of interest. The reviewer PZ declared a past co-authorship with one of the authors CG to the handling editor.
